# The first ITS2 sequence data set of eDNA from honey of Malaysian giant honeybees (*Apis dorsata*) and stingless bees (*Heterotrigona itama*) reveals plant species diversity

**DOI:** 10.1186/s13104-023-06495-9

**Published:** 2023-09-12

**Authors:** Nurul Huda, Saeed ullah, Roswanira Abdul Wahab, Mohd Nizam Lani, Nur Hardy Abu Daud, Amir Husni Mohd Shariff, Norjihada Izzah Ismail, Azzmer Azzar Abdul Hamid, Mohd Azrul Naim Mohamad, Fahrul Huyop

**Affiliations:** 1 Faculty of Sustainable Agriculture, Universiti Malaysia Sabah, Sandakan, Sabah 90509 Malaysia; 2 Department of Biosciences, Faculty of Science, Universiti Teknologi Malaysia, Johor Bahru, 81310 UTM Malaysia; 3 Department of Chemistry, Faculty of Science, Universiti Teknologi Malaysia, Johor Bahru, 81310 UTM Malaysia; 4 Faculty of Fisheries and Food Science, Universiti Malaysia Terengganu, Kuala Nerus, Terengganu, 21030 Malaysia; 5 Greentreebee Enterprise, Kuantan, Pahang, 26070 Malaysia; 6 Department of Biomedical Engineering and Health Sciences, Faculty of Electrical Engineering, Universiti Teknologi Malaysia, UTM Johor Bahru, Johor, 81310 Malaysia; 7 Department of Biotechnology, Kulliyah of Science, International Islamic University Malaysia, Bandar Indera Mahkota, Kuantan, Pahang, 25200 Malaysia

**Keywords:** Honey, ITS2, Metabarcoding, Sequencing, OTU, NCBI

## Abstract

**Objectives:**

Pollen is a useful tool for identifying the provenance and complex ecosystems surrounding honey production in Malaysian forests. As native key pollinators in Malaysia, *Apis dorsata* and *Heterotrigona itama* forage on various plant/pollen species to collect honey. This study aims to generate a dataset that uncovers the presence of these plant/pollen species and their relative abundance in the honey of *A. dorsata* and *H. itama*. The information gathered from this study can be used to determine the geographical and botanical origin and authenticity of the honey produced by these two species.

**Results:**

Sequence data were obtained for both *A. dorsata* and *H. itama*. The raw sequence data for *A. dorsata* was 5 Mb, which was assembled into 5 contigs with a size of 6,098,728 bp, an N50 of 15,534, and a GC average of 57.42. Similarly, the raw sequence data for *H. itama* was 6.3 Mb, which was assembled into 11 contigs with a size of 7,642,048 bp, an N50 of 17,180, and a GC average of 55.38. In the honey sample of *A. dorsata*, we identified five different plant/pollen species, with only one of the five species exhibiting a relative abundance of less than 1%. For *H. itama*, we identified seven different plant/pollen species, with only three of the species exhibiting a relative abundance of less than 1%. All of the identified plant species were native to Peninsular Malaysia, especially the East Coast area of Terengganu.

**Data description:**

Our data offers valuable insights into honey’s geographical and botanical origin and authenticity. Metagenomic studies could help identify the plant species that honeybees forage and provide preliminary data for researchers studying the biological development of *A. dorsata* and *H. itama.* The identification of various flowers from the eDNA of honey that are known for their medicinal properties could aid in regional honey with accurate product origin labeling, which is crucial for guaranteeing product authenticity to consumers.

## Objective

Pollen is a useful tool for identifying the provenance and complex ecosystems surrounding honey production in Malaysian forests. As native key pollinators in Malaysia, *A. dorsata* and *H. itama* forage on various plant/pollen species to collect honey. This study aims to generate a dataset that uncovers the presence of these plant/pollen species and their relative abundance in the honey of *A. dorsata* and *H. itama*. The information gathered from this study can be used to determine the geographical and botanical origin and authenticity of the honey produced by these two species.

## Data description

This dataset contains eDNA sequence information from honey samples of *A. dorsata* and *H. itama*, collected from the East Coast area of Terengganu, Malaysia in June and July 2022. The samples were located at 4° 57’ 6.48” N and 103° 20’ 25.44” E. Individual DNA sequencing and FASTQ files for both samples are available through the National Centre for Biotechnology Information (NCBI) data repository system. The ITS2 nuclear gene region was amplified using previously described primers [1]. The filtered reads were clustered based on k-mer frequency profile using NanoCLUST [2], followed by consensus generation and error correction with Racon and Medaka v.1.4.1 [3].

For *A. dorsata* honey eDNA, a total output of 5 Mb was generated, which assembled into 5 OTUs. For *H. itama* honey eDNA, we obtained 5 contigs with a size of 6,098,728 bp, an N_50_ of 15,534, and a GC content of 57.42. The operational taxonomic unit (OTU) and FASTA file for this sample are accessible via NCBI (https://dataview.ncbi.nlm.nih.gov/object/SRR21831607) (Table 1). For *H. itama*, the raw sequence data shows a total size of 6.3 Mb, assembled into 11 contigs with a size of 7,642,028 bp, an N50 of 17,180, and a GC content of 55.38, based on the NCBI genome annotation pipeline. The operational taxonomic unit (OTU) and FASTA file for this sample are accessible via NCBI (https://dataview.ncbi.nlm.nih.gov/object/SRR21831606).

**Table 1:** General features of *A.dorsata* and *H. itama* predicted by NCBI genome annotation pipeline

Attribute	*A. dorsata*	*H. itama*
Total bases generated (bp)	6,098,728	7,642,028
Number of OTUs identified	05	11
N_50_ (bp) of OTUs	15,534	17,180
G + C content (%) of OTUs	57.42	55.38
Sequence Read Archive	SRR21831607	SRR21831606
BioSample accession	SAMN31155927	SAMN31155926
BioProject accession	PRJNA887189	PRJNA887189

The relative abundance (Ra) of the identified plant and pollen species, along with their taxonomical classification levels (Phylum, Class, Order, Family, Genus, and Species), are presented in Table 2. Each plant species’ individual sequences underwent MEGABLAST analysis to identify highly similar sequences with nearly 100% identity. The complete sequences of selected species were downloaded in FASTA format for subsequent analysis.

The eDNA sequence analysis of honey from *A. dorsata* revealed frequent identification of plant species such as *Corynandra viscosa* (42.02%) and *Syzygium cumini* (40.11%). *C. viscosa*, locally known as Maman pasir, is an erect herb that can reach a height of 1.2 m. It features attractive yellow-colored flowers with a petiole length of 4.5 cm [4]. On the other hand, the genus *Syzygium* comprises over 1,200 species of trees or shrubs with sessile flowers ranging from 7 to 12 cm in height [5]. Every pollen species detected in the honey sample belonged to flowering plants, except for *Mallotus paniculatus* (known locally as Balik Angin), which accounted for less than 1% compared to other flowering plants/pollen species. Additional identified species included *Scaevola taccada* (10.17%), known locally as Merambong, and *Syzygium claviflorum* (7.66%), known locally as Bangkoh. It is worth noting that the identified pollen species in the eDNA sequence are native flowering plants found in the Peninsular Malaysia region where the sample was collected. These species have been previously reported in various studies, such as *C. viscosa* [6], *S. cumini* [7], and *S. taccada* [8].

For *H. itama* the eDNA sequence of honey analysis revealed a significant presence of various plant species. The most abundant species were *M. paniculatus* (Balik angin) (42%) and *Cleome rudisperma* (41%), locally called Maman ungu. *M. paniculatus* is a medicinal plant native to the East Coast of Malaysia [9]. *C. rudisperma*, on the other hand, is a flowering plant reported to be native to Malaysia [10]. Additional plant species identified in the eDNA analysis included *Richardia brasiliensis* (0.53%) [11], *Ludwigia hyssopifolia* (0.42%) (known locally as Lakum air), *Eleucine indica* (0.56%) (known locally as Rumput sambau) [12], *Mimosa pudica* (2.46%) (known locally as Semalu) [13], and *Acacia mangium* (14.49%) (known locally as Manga hutan) [14] (Table 2). Apart from our findings, another study reported a higher abundance of pollen from the phylum Spermatophyta [15]. Specifically, four species, namely *Garcinia oblongifolia, Muntingia calabura, Mallotus pellatus, and Pinus squamata*, were found to occur abundantly and were consumed by *H. itama* in all populations.

**Table 2:** Numbers of plant/pollen species identified from honey samples *A. dorsata* and *H. itama*

Honey sample	Phylum	Order	Family	Pollen/plant species	Ra (%)	Habitat	References
*A. dorsata*	Magnoliophyta	Brassicales	Cleomaceae	*Corynandra viscosa*	42.02	deciduous forests	[4]
	Magnoliophyta	Myrateles	Myrtaceae	*Syzygium cumini*	40.11	secondary rainforest,	[16]
	Spermatophyta	Asterales	Goodeniaceae	*Scaevola taccada*	10.17	coastal forest	[17]
	Magnoliophyta	Myrateles	Myrtaceae	*Syzygium claviflorum*	7.66	terrestrial	[18]
	Magnoliophyta	Malpighiales	Euphorbiaceae	*Mallotus paniculatus*	0.04	secondary rainforest	[19]
*H. itama*	Magnoliophyta	Malpighiales	Euphorbiaceae	*Mallotus paniculatus*	42	Secondary Rainforest	[19] [9]
	Spermatophyta	Brassicales	Cleomaceae	*Cleome rutidosperma*	41	ruderal habitat	[20]
	Spermatophyta	Fabales	Fabaceae	*Acacia mangium*	14.49	coastal tropical lowlands	[21] [22]
	Spermatophyta	Fabales	Fabaceae	*Mimosa pudica*	2.46	terrestrial	[13]
	Spermatophyta	Poales	Poeceae	*Eleucine indica*	0.56	riverside, beaches	[23]
	Magnoliophyta	Myrtales	Rubiaceae	*Richardia brasiliensis*	0.53	any open places	[24] [25] [26]
	Spermatophyta	Myrtales	Onagraceae	*Ludwigia hyssopifolia*	0.42	wetlands	[27]

Note: Ra: Relative abundance or percentage of pollen based on plant species foraged by *Apis dorsata* and *Heterotrigona itama*

### Limitations

Sample size: A small sample size may not be representative of the larger population and may limit the generalizability of the findings.

Regional specificity: The study focuses on honey samples from the Peninsular Malaysia region, which may limit the generalizability of the findings to other regions or countries.

Identification methods: The study uses eDNA sequencing and pollen analysis to identify plant species in the honey samples.

Honey production: honey was collected from multiple hives in one area. This could affect the diversity and abundance of plant species present in the honey samples.

Honey age: The age of honey can affect the diversity and abundance of plant species present in the sample.

## Data Availability

https://www.ncbi.nlm.nih.gov/sra/SRX17820767. https://www.ncbi.nlm.nih.gov/sra/SRX17820766. https://www.ncbi.nlm.nih.gov/sra/SRX17820765.
